# Surfactant-driven modulation of protein corona on solid lipid nanoparticles: Insights including molecular docking studies

**DOI:** 10.1186/s40360-025-01017-8

**Published:** 2025-11-03

**Authors:** Shelly Roselyn Van Eyssen, Doğa Kavaz

**Affiliations:** Biotechnology Research Centre, Cyprus International University, Nicosia, Northern Cyprus, Via Mersin 10, 99258 Turkey

**Keywords:** Protein corona, Solid lipid nanoparticles, Surface charge, Protein adsorption, Drug release, Molecular docking

## Abstract

**Background:**

Nanoparticles have been used in nanomedicine for therapeutic purposes. Lipids have been a popular choice of material in the synthesis of biocompatible nanoparticles due to their ability to encapsulate diverse therapeutic agents. However, once introduced into biological environments, nanoparticles rapidly adsorb proteins on their surface, forming a protein corona that can alter drug release, targeting and cellular interactions. Understanding these effects is essential for optimizing solid lipid nanoparticles (SLNs) as drug delivery systems.

**Methods:**

SLNs were synthesized using stearic acid (SA) as the lipid core and stabilized with surfactants, Tween 80 and SDS (sodium dodecyl sulphate). To mimic in vitro conditions, SLNs were incubated with 5% FBS (foetal bovine serum) for varying durations to allow soft and hard protein corona formation. SLNs were characterized for size and surface charge via dynamic light scattering while UV visible spectroscopy, protein adsorption studies, FTIR and the Bradford Assay were employed to confirm and quantify protein corona formation. In parallel, molecular docking was conducted to analyse protein-nanoparticle interactions. To investigate the potential influence of protein corona formation on drug release, ceftriaxone (CTX) was encapsulated within SLNs followed by immediate incubation to allow corona formation. Subsequent evaluations included drug leakage, release profile and antibacterial efficacy against *Staphylococcus aureus* and *Pseudomonas aeruginosa*.

**Results:**

Protein adsorption was confirmed on all SLN formulations and closely correlated with nanoparticle surface charge. Formulations with more negative zeta potentials adsorbed higher amounts of protein. SDS concentration influenced protein adsorption with higher SDS content reducing overall corona formation. Molecular docking revealed that protein-ligand interactions were maintained by hydrogen bonding and hydrophobic forces. Drug release studies confirmed that the protein corona reduced cumulative drug release by 6%. Antibacterial assays confirmed slightly reduced efficacy against both strains aligning with the reduced cumulative drug release.

**Conclusions:**

This study demonstrated that protein corona formation on dual-surfactant SLNs is strongly influenced by surface charge and surfactant composition and in turn modulates drug release dynamics. The protein corona was shown to modulate drug release with potential implications for antibacterial efficacy. These findings highlight the importance of tailoring surface properties to control protein-nanoparticle interactions thus providing insight into the design of lipid-based nanoparticles for therapeutic applications.

## Background

Solid lipid nanoparticles (SLNs) have been in use as pharmaceutical nanocarriers since 1996, as approved by the FDA [1]. Amongst the various medicinal fields of application, drug delivery of antibiotics has been a branch of interest. Solid lipid nanoparticles exhibit a myriad of favourable properties ranging from high drug encapsulation [2], good drug release kinetics [3], biocompatibility [4], biodegradability [5] etc. Within the context of microbiology, solid lipid nanoparticles have been identified to reduce antimicrobial resistance (AMR) via reduction in biofilm formation [6], improving cellular uptake [7], reduction in pump mediated drug expulsion [8] and bypassing the effect of antibiotic-modifying enzymes [9]. SLNs are usually surfactant stabilized which in turn enables them to be solid at even room temperature [10]. The surfactant also plays a role in reducing surface tension and enhancing drug encapsulation. Solid lipid nanoparticles are commonly synthesized with one surfactant [10] however, synthesis with two surfactants has proved advantageous [11].

Ceftriaxone (CTX) is a third generation cephalosporin whose anti-bacterial efficacy almost 30 years ago cut across even antibiotic resistant strains [12]. In the early 2010s it was noted that ceftriaxone was losing its potency against even the microbial strains previously susceptible to the drug [13, 14]. In an endeavour to thwart antimicrobial resistance (AMR), the use of pharmaceutical carriers in antibiotic delivery has been explored over the past years. In particular, pharmaceutical nanocarriers have presented a new avenue in the fight against multiple drug resistant (MDR) bacteria [15]. Nanocarriers protect antibiotics like ceftriaxone from enzymatic degradation by bacterial enzymes prior to therapeutic action [16] and facilitate penetration through the biofilm matrix [17]. Whilst they can be used on their own as antibacterial agents to combat AMR, pharmaceutical nanocarriers (nanoparticles) have been used as drug delivery agents, hence the term ‘carrier’ [18]. Combining both the potential antibacterial efficacy of the carrier and the drug, a promising solution to AMR can be attained.

In the recent years, researchers have investigated the behaviour and interactions of nanocarriers in vitro and in vivo. The formation of a biofilm comprised of lipids, sugars but mainly proteins around the surface of these nanocarriers, dubbed the protein corona, has been well documented [19]. This protein corona can be both advantageous and disadvantageous with regards to the intended use of the nanocarrier. The protein corona can enable cellular uptake and boost hemocompatability in organisms [20, 21]. On the other hand, the formation of a thin layer of protein around the surface of the nanocarriers can inhibit the interaction of the nanocarrier and the surface of the bacteria in the case of AMR and also alter the drug release profile of the nanocarrier [22]. In vitro studies involving the protein corona are important as they provide a better control of the numerous variables associated with protein corona formation thereby enabling more precise mechanistic insights that can inform correlation with in vivo outcomes [23]. For preliminary physicochemical investigations, bovine serum albumin (BSA) is frequently employed as it is cost-effective and structurally comparable alternative to human serum albumin (HSA) as they share 76% identity [24].

Bioinformatics, an ever-evolving field, has allowed us to better understand the interaction mechanisms on a molecular level. Molecular docking, an in silico process, uses computational visualizations to view protein-ligand interactions [25]. In the present work, molecular docking was employed to corroborate experimental observations of protein corona formation on SLNs.

This study investigates the formation of the protein corona on SLNs synthesized from stearic acid using two surfactants. While SLNs are conventionally prepared with a single surfactant [26], dual surfactant systems have been shown to improve stability e.g., sodium dodecyl sulphate (SDS) can increase nanoparticle stability in suspension. Following protein corona formation, the effect of ceftriaxone delivery was evaluated against two bacterial strains: *Staphylococcus Aureus* and *Pseudomonas Aeruginosa*.

## Methods

### Preparation of solid lipid nanoparticles (SLN), ceftriaxone-loaded solid lipid nanoparticles (CTX-SLN) and ceftriaxone loaded solid lipid nanoparticles with protein corona (FBS-CTX-SLN)

A total of five distinct formulations of solid lipid nanoparticles were prepared as outlined in Table 1using the solvent emulsification technique. 100 mg of solid stearic acid was dissolved in 2 ml of ethanol at 70 °C to form the lipid phase. The required quantities of Tween 80 and sodium dodecyl sulphate (SDS), as specified in Table 1, were measured and dissolved in 25 ml of distilled water to constitute the aqueous phase. The lipid phase was introduced to the aqueous phase surfactant solution dropwise under homogenization at 12,000 rpm for 10 minutes. To synthesize ceftriaxone loaded solid nanoparticles (CTX-SLN), 1 ml of a 10 mg/ml ceftriaxone solution was incorporated in the 25 ml distilled water containing Tween 80 and SDS, yielding an aqueous phase with a final drug concentration of 0.4 mg/ml. The lipid phase was subsequently added dropwise to the drug-surfactant aqueous phase under identical homogenization conditions. Both unloaded and drug loaded solid lipid nanoparticles were cooled in an ice bath for 10 minutes, sonicated for a further 10 minutes and centrifuged for 15 minutes at 8000 rpm. To obtain protein corona modified CTX-SLNs (FBS-CTX-SLNs), freshly prepared CTX-SLNs were incubated with 5% FBS for 30 minutes at 37 °C and centrifuged at 6000 rpm.

**Table 1:** The different ratios of Tween 80 and SDS used in the synthesis of stearic acid nanoparticles. Stearic acid amount and the speed of homogenization was kept constant

Formulation code	Tween 80 concentration (%v/v)	SDS concentration (%w/v)	Stearic acid amount (mg)	Homogenization speed (rpm)
SLN-1	0.5	0.5	100	11000
SLN-2	0.75	0.5	100	11000
SLN-3	1	0.5	100	11000
SLN-4	0.5	0.75	100	11000
SLN-5	0.5	1	100	11000

### Particle size and zeta potential

The size and zeta potential of all five formulations of nanoparticles were analysed using the Malvern ZTS1240. The results of the size and zeta potential are represented mean of two measurements run twice.

### FTIR

To confirm protein corona formation, the functional groups of stearic acid solid lipid nanoparticles (SLN), foetal bovine serum (FBS), and protein corona modified solid lipid nanoparticles (FBS-SLN) were identified using Fourier Transform Infrared Spectroscopy (Shimadzu).

### Ultra-violet visible (UV-Vis) spectrophotometry

To examine the time progression of the interaction of protein with the solid lipid nanoparticles, UV-Vis spectrophotometry was performed using an Ultra-violet Visible Spectrophotometer (Shimadzu, Japan).

### Interaction mechanism

To be able to analyse the content of both Tween 80 and SDS present in the lipid nanoparticles, a UV-Vis spectrophotometry method was used [27]. Calibration curves for both Tween 80 and SDS were prepared. The solid lipid nanoparticles were ultra-sonicated in water for a period of five minutes and filtered through a 0.45 µm Whatman filter. The absorbances were measured at 243 nm and 650 nm for Tween 80 and SDS respectively. The content of Tween 80 and SDS in the nanoparticles was then calculated.

### Incubation with foetal bovine serum (FBS)

The five different formulations of stearic acid nanoparticles were each incubated in 5%FBS concentration dissolved in Dulbecco’s Modified Eagle Medium (DMEM) media to mimic cellular like conditions in in vitro experiments. The incubation times were 1 hr, 6 hr, 24 hr and 48hrs shaking at 100rpm at 37 °C.

### Protein corona elution and protein content analysis

After incubation, the nanoparticles in protein solution were centrifuged at 13000rpm for 10 minutes. The nanoparticles were washed twice in PBS (pH 7.4). The protein corona was eluted using a 1 M Tris-HCl-SDS solution at pH 7.4. The eluted protein was analysed for total protein content using the Bradford 96-well plate Assay.

### Protein adsorption

The main protein present in FBS is bovine serum albumin (BSA). To assess the mechanism of protein adsorption on the surface of the nanoparticles, all five formulations of nanoparticles were incubated with BSA. Different concentrations of BSA ranging from 1 to 100 µM were prepared and incubated with each formulation of nanoparticle for 45 minutes. The hydrodynamic sizes of the nanoparticles before and after incubation were measured and by using the Hill equation, the binding affinities and Hill co-efficient were determined.

### In silico evaluation

To further analyse the binding interaction between the main protein present in FBS, bovine serum albumin (BSA), and the two surfactants, Tween 80 and SDS, a molecular docking approach was used. BSA with PDB ID 3V03 was downloaded from the Protein Data Bank [28]. Docking was performed with AutoDock Tools version 1.5.7. Prior to the docking procedure, the protein was prepared by removing water molecules, adding polar hydrogens and Kollman charges were assigned [29]. The ligand structures were downloaded from PubChem, Tween 80 (PubChem SID 481110309) and SDS (PubChem CID 3423265). Docking was performed using the default parameters and a large grid box (126 ×126 x 126) with a grid box spacing of 0.519Å to allow free movement around the protein. To analyse the ligand-protein interactions further, Pymol version 3.1.1. was used.

### Drug encapsulation, drug loading and drug leakage

Ceftriaxone (CTX) presents a maximum absorbance at 241 nm via spectrophotometry. A calibration curve of the drug was constructed. Immediately after synthesis, the drug loaded nanoparticles were centrifuged at 8000 rpm. To quantify the amount of ceftriaxone encapsulated by the solid lipid nanoparticles, the supernatant of the CTX-loaded nanoparticles was collected and the absorbance at 241 nm was measured (Nanophotometer, Implen). This absorbance will determine the amount free drug present in the solution. In order to calculate the amount of ceftriaxone encapsulated in the solid lipid nanoparticles, the following formula was used:

$${\rm{\% }}drug\,encapsulated = {{total\,drug - free\,drug} \over {total\,drug}}\times100$$

The drug loaded nanoparticles were dried and weighed to quantify the drug loading. Drug loading expresses the proportion of the drug’s weight relative the drug-loaded system and is calculated using the following formula:

$${\rm{\% }}drug\,loading = {{weight\,of\,entrapped\,drug} \over {weight\,of\,nanoparticles}}\times100$$

An investigation on drug leakage on CTX-loaded lipid nanoparticles and FBS-CTX-lipid nanoparticles was performed by suspending the solid lipid nanoparticles in distilled water and stirring rapidly for 15 minutes. The samples were then centrifuged at 8 000rpm and the supernatant analysed for drug content via spectrophotometry.

### In vitro drug release

The in vitro release was evaluated using the dialysis bag method for 72 hours. A 30kDa was loaded with CTX-loaded nanoparticles and FBS-CTX-loaded nanoparticles was dissolved in 1X PBS (pH 7.4) and placed in a beaker containing 1X PBS (pH 7.4). (Separate dialysis bags and beakers were used). The beaker was subjected to agitation of 100rpm at a temperature of 37 °C. At established time intervals, 1 ml of solution from the beaker was pipetted from the beaker and replaced with fresh PBS solution. The pipetted solution was analysed for drug content. The drug content at the established time intervals was used to construct in vitro release graphs. The cumulative release data was also utilized to analyse the drug release kinetics.

### Kirby-Bauer test

One colony of each of the two strains of bacteria, *Staphylococcus Aureus* and *Pseudomonas Aeruginosa*, were cultured overnight in nutrient broth at 37 °C. The bacteria were normalized at 600 nm to OD 0.1 and calculated amounts were spread across Mueller-Hinton agar plates. Discs containing 10 µl of the SLN, SLN-CTX and FBS-SLN-CTX formulations dissolved in PBS, PBS (negative control) and ceftriaxone (positive control) were placed equidistant on the agar plates. The plates were incubated overnight and zones of inhibition were measured and recorded.

## Results and discussion

### Size and zeta potential

Table 2 shows the size, zeta potential and polydispersity index (PDI) of the five formulations of nanoparticles. It can be noted that all five formulations synthesized nanoparticles of 270 nm and below. When the least amount of SDS was used (0.5%w/v), the nanoparticle sizes were the smallest (155 nm to 204 nm). However, maintaining the lowest concentration of Tween 80 resulted in nanoparticles of a larger size ( > 250 m). It was noted that combining equal concentrations of Tween 80 and SDS produced nanoparticles with a size < 200 nm and the lowest surface charge of −36.2 mV. Doubling the concentration of Tween 80 compared to SDS as compared to doubling concentration of increased the size of formulated SLN nanoparticles by +60 nm. The same formulation combination appeared to have little effect on the surface charge of the SLN nanoparticles. The highest surface charge (−21.56 mV) was synthesized via a formulation that contained the highest concentration of Tween 80 (1%).

**Table 2:** Stearic acid formulations and their corresponding sizes, zeta potential and polydispersity index (PDI). The data shown is presented as the mean±SD where *n* = 3

Formulation code	Size (nm)	Zeta potential (mV)	PDI	
SLN-1	198.3 ± 0.98	−36.2 ± 0.07	0.4609	
SLN-2	155 ± 1.01	−30.97 ± 0.12	0.4767	
SLN-3	204.8 ± 0.87	−21.56 ± 0.31	0.5141	
SLN-4	274.9 ± 1.24	−24.56 ± 0.14	0.5036	
SLN-5	264.8 ± 0.96	−23.28 ± 0.09	0.4980	

### Size and zeta potential changes after incubation

**Fig. 1:**
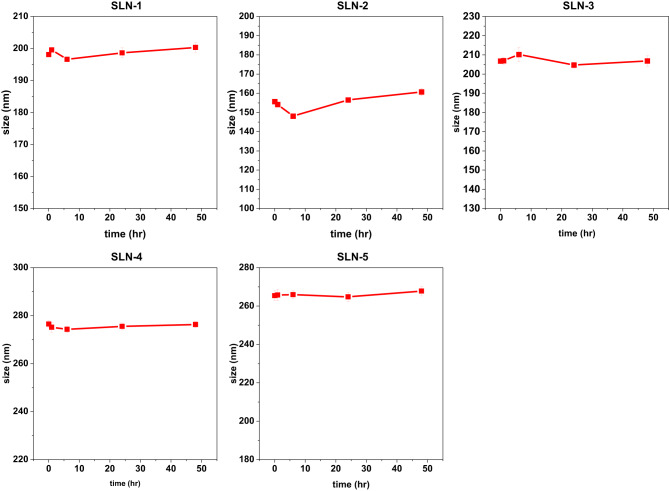
Size changes of nanoparticles after 48hrs of incubation with 5% FBS. Data is presented as mean size change ±SD (*n* = 3)

To assess particle size change due to aggregation or other factors, particle size was measured after protein corona formation. Mean particle size change was measured at 0 h and after incubation with 5% FBS at 1 h, 6 h, 24 h and 48 h. All formulations except S2 had an average mean particle size change of ±2% throughout the 48 h of incubation however S2 showed ±6% mean size change during incubation as seen in Fig. 1.

**Fig. 2:**
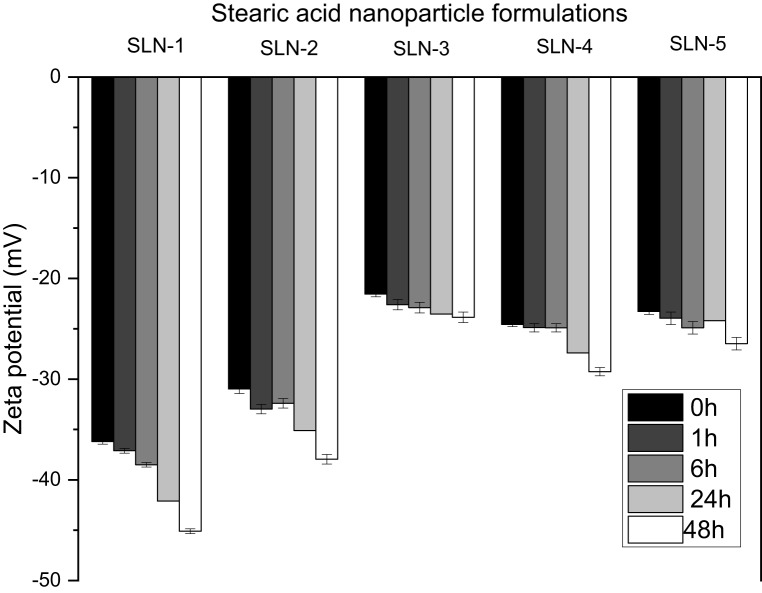
Surface charge changes of stearic acid nanoparticles after incubation in DMEM supplemented with 5% FBS at 0 h, 1 h, 6 h, 24 h and 48 h of incubation. Error bars indicate the standard error calculated from three repeats

Zeta potential was measured at 5 different intervals; 0 h, 1 h, 6 h, 24 h and 48 h of incubation in DMEM supplemented with 5% FBS. The results showed a decrease in zeta potential in all formulations (Fig. 2). The most pronounced decrease was observed for SLN-1 which initially displayed the lowest surface charge (−36.2 mV). After 48 h SLN-1 demonstrated an 8.9 mV reduction in surface charge corresponding to an overall 24.5% decrease in surface charge. In contrast, SLN-3, the nanoparticle formulation that has the highest initial surface charge of the 5 formulations (−21.56 mV) exhibited a 9.3% overall reduction. An average minor decrease of 2.3% in surface charge was recorded for formulations SLN-2, SLN-4 and SLN-5.

### Colloidal stability of stearic acid nanoparticles after 90 days

**Fig. 3:**
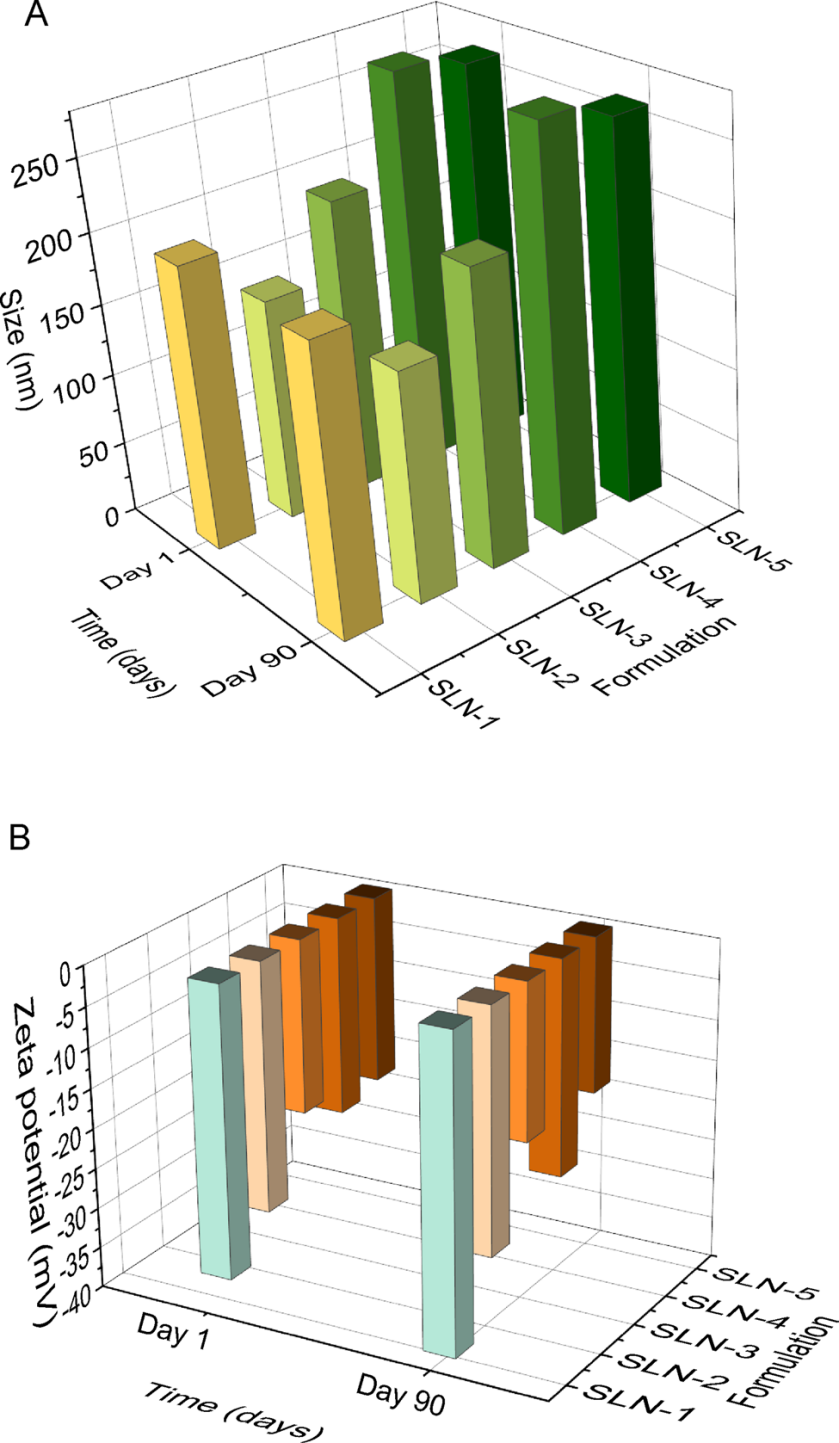
A shows the mean sizes (*n* = 3) on day 1 and after 90 days of storage of the 5 formulations (SLN-1 to SLN-5). B shows the mean zeta potential (*n* = 3) after 90 days of storage of the 5 formulations (SLN-1 to SLN-5). The lipid nanoparticles were stored at 4 °C

Figure 3 shows the mean sizes and zeta potentials measured on day 1 and after 90 days of storage in darkness and at a temperature of 4 °C. Dynamic light scattering revealed negligible size variations across all five lipid formulations over the 90-day period, with changes ranging from −1.9 nm to +1.1 nm. The zeta potential changes were minimal with average variations between −3.96 mV and +2.22 mV. Storing lipid nanoparticles above freezing temperatures has proven suitable for the type of nanoparticle resulting in stability. A study by [30] found that lipid nanoparticles stored above 0 °C versus those stored at −20 °C were more stable over a 150-day period. Another recent study found that storing lipid nanoparticles between 2 and 5 °C was the optimum temperature to promote colloidal stability [31]. Therefore, all five formulations synthesized in this study exhibit colloidal stability. The colloidal stability of formulations over a lengthy period can be awarded to the inclusion of SDS as an anionic surfactant [32].

### FTIR

**Fig. 4:**
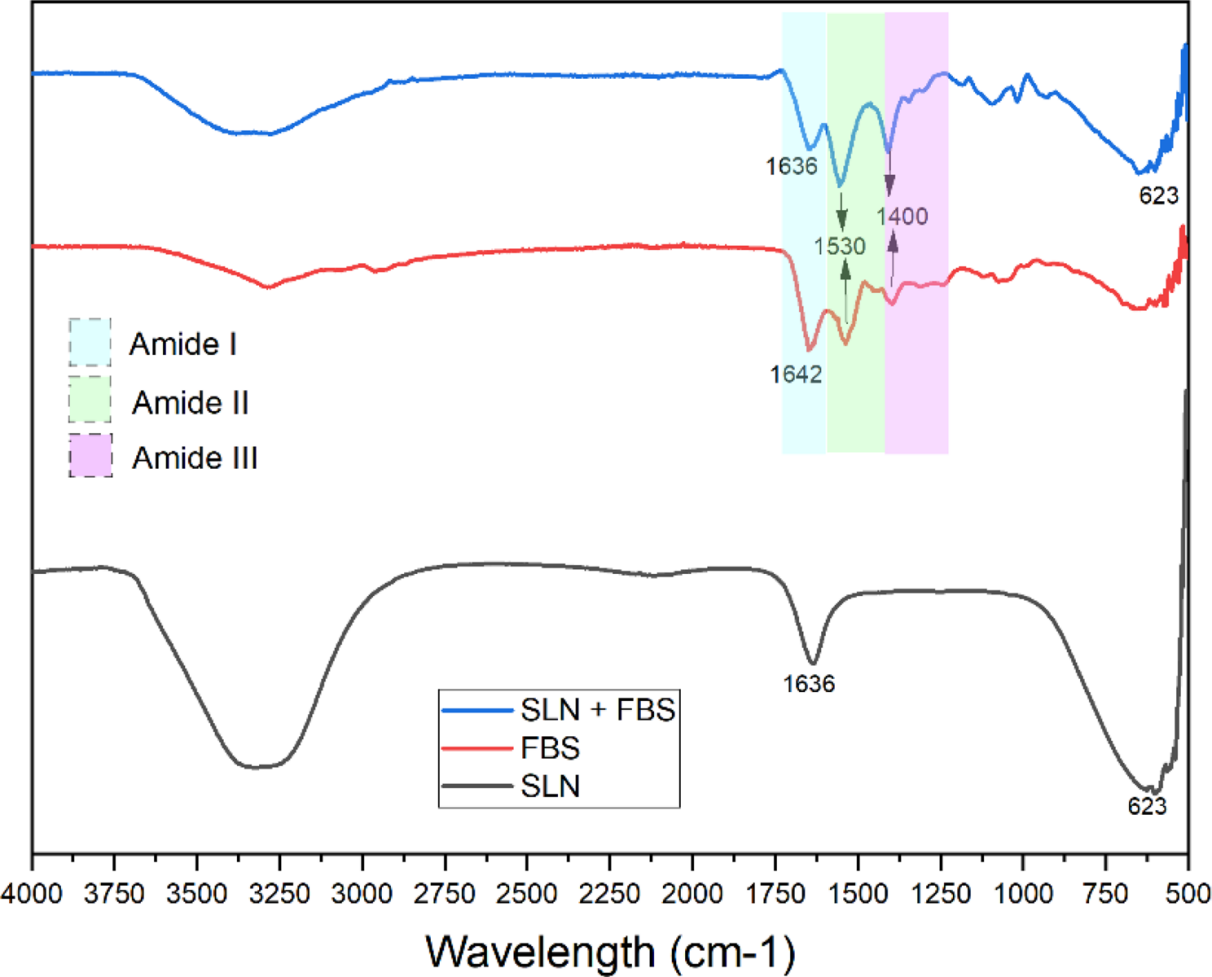
FTIR spectra of stearic acid nanoparticles

To analyse formation of stearic acid solid lipid nanoparticles and to confirm the possible interactions of the protein on the surface of the nanoparticles, FTIR analysis was performed, and the IR spectra were shown on Fig. 4. The characteristic broad peak between 3500 cm^−1^ and 3000 cm^−1^ represents the OH group present on the structure of the stearic acid nanoparticles [33]. The weak peak at 2123 cm^−1^ is indicates the possibility of alkene groups (triple C bonds) in the nanoparticle structure [34]. At 1636 cm^−1^, a distinct peak is seen representing the C = O bonds that is also present in the structure stearic acid. The C-C bonds in the backbone structure of the stearic acid are indicated at the 632 cm^−1^ stretch [35]. The peak is also indicative of a C-H bend. To confirm the interaction of FBS with the nanoparticles, the FTIR spectra of the FBS and the stearic acid nanoparticles after incubation with FBS (SLN + FBS) were also determined. The FTIR spectra of FBS allows the characterization of the protein secondary structure. The secondary protein structures are found in the wavelength regions of 1700-1600 cm^−1^ (amide I group), ~1550 cm^−1^ (amide II group) and ~1300 cm^−1^ (amide II group) [36–38]. The peak at present ~1640 cm^−1^in all 3 spectra shows that subsequent protein-nanoparticle interaction, specifically with the amide I group, which is associated with the C = O stretching vibration present in the backbone of peptides [39]. The peak at ~1640 cm^−1^ is also indicative of β-sheet protein secondary structures (X. Wang et al., 2017). The peak at 1530 cm^−1^ present in both the FBS and SLN + FBS spectra in the amide II region confirms the presence of the N-H bending vibration [39]. The peaks in the region between 1400 cm^−1^ and 1300 cm^−1^ pinpoint towards the amide III region, specifically indicating α-helices [40] and an N-H bending vibration at 1400 cm^−1^.

### UV-Vis absorption spectra

**Fig. 5:**
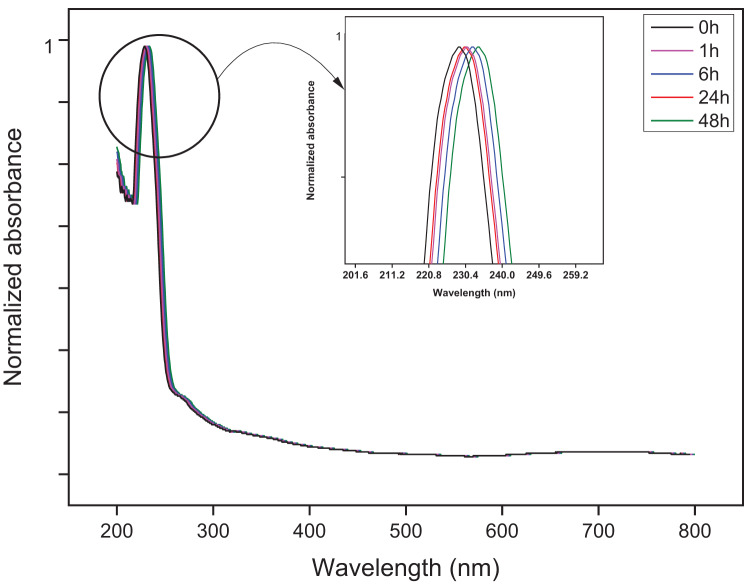
Normalized UV-Vis spectra of the solid lipid nanoparticles after intervals of incubation with 5% FBS. All five formulations exhibited the same spectra

To further characterize the SLN-FBS complexes formed, and to asses potential alterations formed in the nanoparticles, i.e., protein and nanoparticle interaction, UV-Vis spectroscopy was performed. Absorbance values recorded and normalized to an optical density of 1. Figure 5 shows that expected slight shift to the right caused by structural changes that occur on the nanoparticle surface as protein corona formation occurs with a maximum peak detected at 231 nm. UV-Vis spectrophotometry a common technique used to confirm the presence of adsorbed protein on the surface of nanoparticles and for detecting potential ultra-violet induced changes that may occur within the proteins as they form a corona (Mishra et al., 2021). All five formulations presented a similar spectrum shown in Fig. 5. At 213 nm, a maximum peak was observed coinciding with previously reported spectra of SLN nanoparticles [41]. The spectra indicated, by the slight right shift, the hardening of the protein corona after 48 h. Aside from analysing protein-nanoparticle complex formation, UV-Vis spectra also verified the absence of nanoparticle aggregation before and after incubation.

### The interaction of proteins with tween 80 and SDS

The Tween 80 and SDS content were quantified using UV-Vis spectroscopy method and the results are tabulated in Table 3. The amounts of Tween 80 and SDS used in the synthesis (Table 1) of the solid lipid nanoparticles correspond closely with the amounts present in the lipid nanoparticles indicating efficient incorporation of the surfactants onto the nanoparticles. Roughly 10% of the Tween 80 in the synthesis solution was retained in the synthesized nanoparticles. The presence of Tween 80 contributes to the hydrophilic character of the nanoparticle surface. Literature reports have found that a hydrophobic surface of a nanoparticle will encourage protein-nanoparticle interactions as compared to a hydrophilic surface [42]. A hydration layer that forms on the surface is said to be the preventative barrier in protein adsorption [43]. For the SDS, ~ 20% of the initial synthesis quantity was present in the formulated lipid nanoparticles. In the present study, the level of protein adsorption observed for each nanoparticle formulation correlated with the SDS content thereby suggesting a potential influence of SDS concentration on protein corona formation.

**Table 3:** Calculated amounts of Tween 80 and SDS present in the formulated solid lipid nanoparticles

Formulation	Content of Tween 80(%v/v) ±SD	Content of SDS(%w/v) ±SD
SLN-1	0.0231±0.43	0.0852±0.34
SLN-2	0.0671±0.04	0.1054±0.28
SLN-3	0.10297±0.17	0.1154±0.15
SLN-4	0.0193±0.32	0.1425±0.03
SLN-5	0.0263±0.07	0.1871±0.45

### Protein adsorption

The generally implemented strategy in research pertaining to the study of protein corona formation on nanoparticles is to expose the nanoparticle of interest to a biological fluid, harvest the nanoparticle from the fluid and eventually study the adsorbed layer. The processes that lie in between the harvest and study of the layer tend to be abrasive and rough e.g., centrifugation at very high speeds. This is done to ensure the protein corona is made of only those proteins which are bound onto the surface of the nanoparticle and all unbound protein is not included in the analysis.

To largely explain the adsorption of proteins on the surface of nano-sized particles, the Hill Equation [44] is employed. The Hill Eq. (1) states that the surface coverage of the nanoparticle by protein (θ) is dependent on the concentration of the protein [P] which gives rise to the Hill coefficient (n) and the dissociation constant (K’_D_):

1 $$\theta = {{{{\left[ P \right]}^n}} \over {{{\left[ P \right]}^n} + K{^{\prime}}{D^n}}}$$

The dissociation constant conveys where the protein concentration is at half coverage (50% of the binding sites are occupied; quantifying the strength of the NP-protein interface. The Hill is a measure of the steepness of the curves constructed and consequently explains the manner of cooperativity binding. For binding to be considered non-cooperative, binding is independent to the number of ligands bound to the protein, and the *n* value is 1 [45]. When the *n* value is greater than 1, there is positive cooperative binding whilst an *n* value less than one indicates the opposite. Relating the hydrodynamic size of the nanoparticles (d_z_) and their subsequent hydrodynamic change in size (Δ*d*_*z*_) to the Hill equation gives rise to the following equation:

2 $${d_z}[P] = {d_z}{(0)^3}\sqrt {{{1 + c\, \cdot \,{{[P]}^n}} \over {{{[P]}^n} + {{({{K{^{\prime}}}_D})}^n}}}} $$

**Fig. 6:**
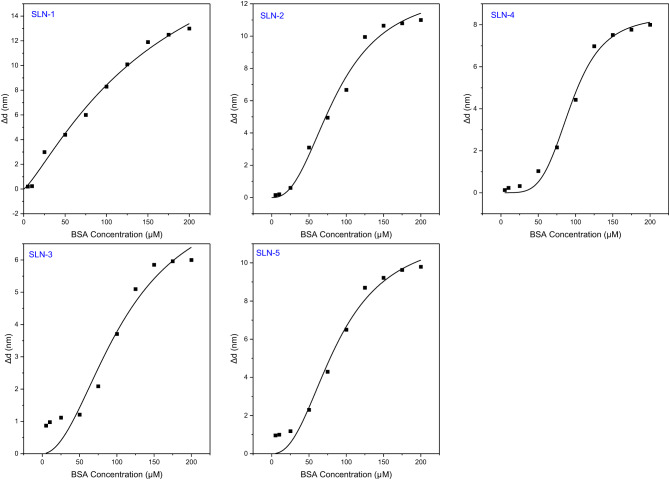
DLS curves relating diameter change and BSA concentration using the hill Eq. (2)

In this equation *dz*[P] and *dz*(0) are the hydrodynamic sizes of the nanoparticles after and before incubation with BSA respectively and c being the scaling constant. The curves fitting Eq. (2) are shown in Fig. 6 and the values of n and K’_D_ are shown in Table 4. For all the lipid nanoparticle formulations, the *n* values were all larger than one. This indicates that the manner of cooperative binding between the protein and nanoparticle was positive. The positive *n* value indicates a possible multiple interaction sites to the lipid nanoparticles [46]. It also indicates that when BSA binds to the lipid nanoparticles, its affinity towards the nanoparticle increases linearly [46]. A Hill coefficient greater than one also infers that multiple layers of BSA form on the surface of the nanoparticles [47]. The nanoparticle formulation SLN-3 showed an *n* value close to 1 which suggest non-cooperative binding and coincidentally represent the formulation with the highest SDS concentration.

**Table 4:** Protein adsorption parameters on SLN lipid nanoparticles

Nanoparticle formulation	K’_D_	n	R^2^
SLN-1	163.8	1.23	0.9908
SLN-2	87.9	1.42	0.9903
SLN-3	109.5	1.03	0.9360
SLN-4	94.2	1.32	0.9920
SLN-5	88.3	1.30	0.9780

### In-silico evaluation

**Table 5:**
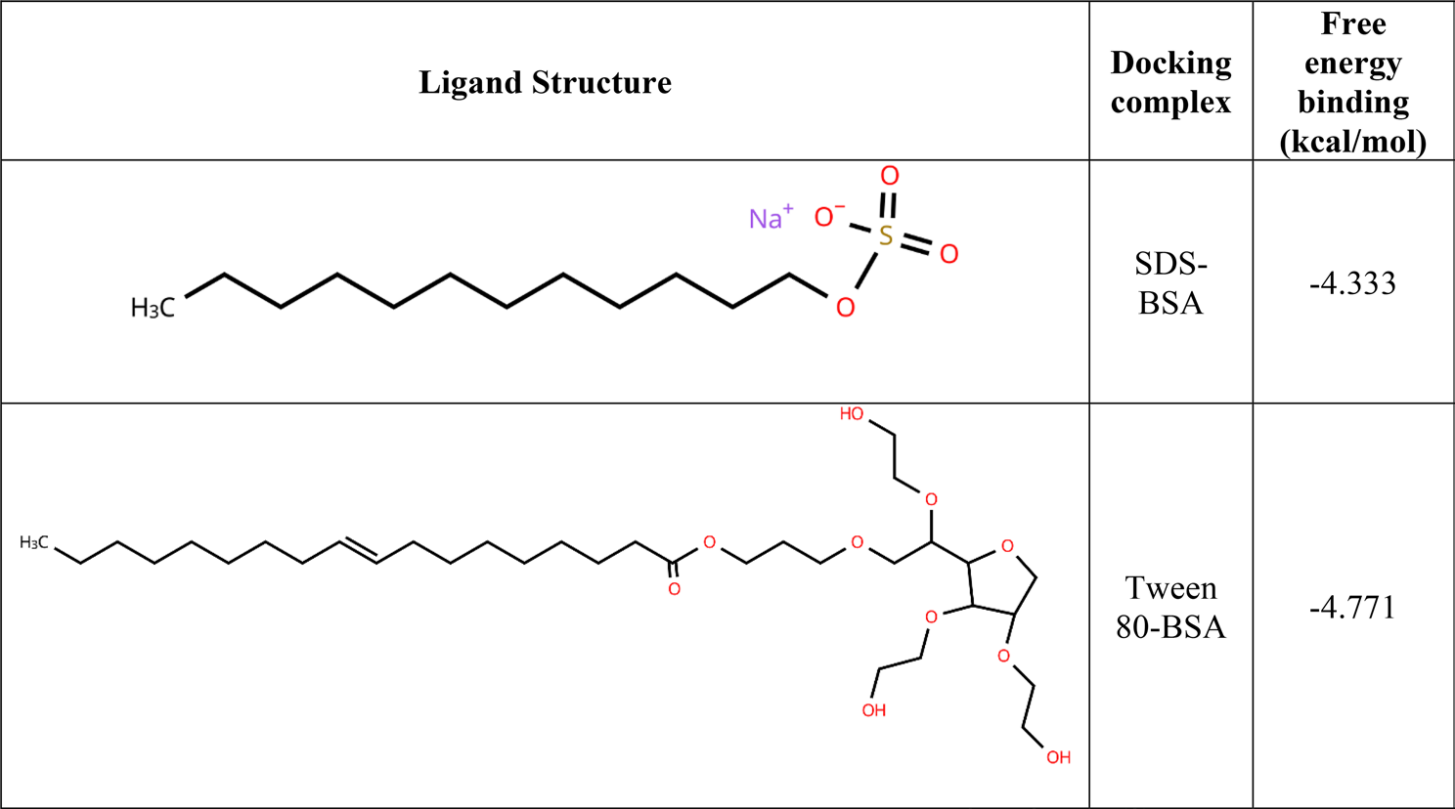
The results of the docking complexes for the bovine serum albumin complexes based on affinity including structures of the ligand

Molecular docking assisted with understanding the strength of the binding affinities of the surfactants to the protein. Molecular docking calculated free energy binding values measured in kcal/mol. Docking simulations results that are greater than 0 kcal/mol indicate an either low or lack of binding affinity for BSA [48]. A binding affinity less than 0 kcal/mol is considered to represent the opposite; medium-high binding affinity. The two ligands in this study (surfactants) both presented binding energies less than 0 kcal/mol as seen in Table 5. This points to a favourable interaction between both ligand and protein.

**Fig. 7:**
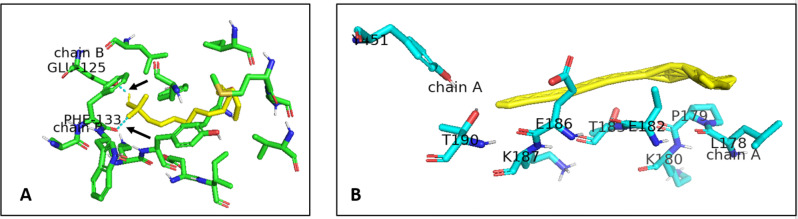
Binding site residues: (**A**) - SDS-BSA complex and (**B**) - Tween 80-BSA complex. Both the BSA residues and the surfactant were represented as sticks. Black arrows show the polar interactions indicated by cyan dotted lines. The surfactants in both Fig. **A** and **B** are shown in yellow

Figure 7 shows the amino acid residues located in the binding pockets of BSA for both surfactants at the radial distance of 5Å. This radius ensures all types of bonds – hydrogen and van der Waal’s alike - are included in the analysis. As seen, in Fig. 7A, the polar head of sodium dodecyl sulphate interacts directly with GLU-125 (polar) and PHE-133 (non-polar) in the Domain IIB, whilst the hydrophobic chain is seen to interact with other residues namely, LEU-115, LEU-122, GLU-125,LYS-132, PHE-133, LYS-136, TYR-137, ILE-141, TYR-160, ILE-181, MET-184, ARG-185 and VAL-188. This the case with the tail of SDS having intramolecular interactions with residues [49]. The Tween 80-BSA interaction was made up of non-polar interactions within the Domain IIA. Both the tail and head interacted with the residues LEU-178, PRO-179, LYS-180, GLU-182, THR-183, GLU-186, LYS-187, THR-190 and TYR-451 shown clearly in the binding pocket in Fig. 7. The main mode of interaction between Tween 80 and the BSA residues is via hydrophobic interactions as opposed to ionic surfactants which interact via hydrogen bonding [50].

### Protein content analysis

**Fig. 8:**
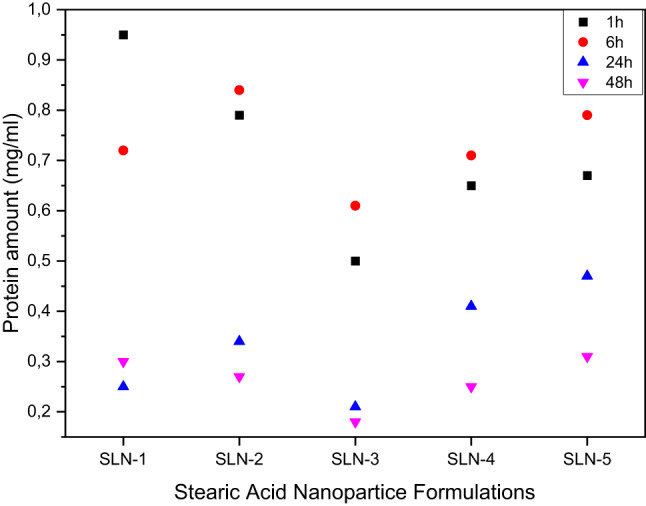
Total protein content harvested by elution from the surface of the solid lipid nanoparticles over the 48 h period at 1 h, 6 h, 24 h and 48 h intervals. The results plotted are mean values of three different trials

The total protein content eluted off the nanoparticle surface was quantified using the Bradford 96 well plate Assay. The results shown in Fig. 8 illustrate that across all nanoparticle formulations, a larger protein content was present in the protein corona in the 1 h and 6 h incubation times compared to the 24 h and 48 h incubations times. Maximum protein adsorption occurred after 1 h & 6 h incubations with a decline after 24 h & 48 h. This is difference attributed to the ‘soft corona’ and ‘hard corona’ phenomenon. Proteins making up the soft corona form weak and non-permanent bonds between them and the nanoparticle surface whilst the hard corona establishes more permanent bonds with the nanoparticle surface [51]. During the 1 h & 6 h incubation times, proteins from both the ‘soft corona’ and ‘hard corona’ resulted in a higher total protein content. Among the five formulations, SLN-1 exhibited the highest protein adsorption with a concentration of 0.95 mg/ml which decreased to 0.3 mg/ml after 48 h; a 63% reduction. SLN-2, SLN-4 and SLN-5 followed a similar pattern and averaged a 40% decrease in protein content between 1 h and 48 h. In contrast, SLN-3 consistently adsorbed the least protein with a maximum of 0.61 mg/ml at 6 h which dropped to 0.18 mg/ml by 48 h.

The relationship between surface charge and total protein present in the protein corona was evident in these results. SLN-1 which exhibited the lowest surface charge adsorbed more protein on its surface as compared to SLN-3 which has a comparatively higher surface charge. The results of the total protein content tie in with the fact that there is an established link between negative surface charge and more protein corona formation [52, 53]. Another study also reported a −19 mV decrease in zeta potential by adsorption of proteins on the surface of negatively charged solid lipid nanoparticles [54]. The presence of stearic acid, a fatty acid containing a carboxylic group, along with the surfactant used in the formulation contributes to this negative surface charge. Negatively charged nanoparticles adsorb more two to three times more protein than positive nanoparticles. The composition of the protein corona found in negatively charged protein corona are comprised of opsonins and dysopsonins. This enhanced adsorption may present drawback as negatively charged nanoparticles often display lower cellular internalization compared to the positively charged nanoparticle systems [55]. Thus, to facilitate cell up-take without use of surface loaded ligands, the protein corona can be used to increase the rate of cell up-take of SLNs by pre-coating [56]. Furthermore, the protein corona will also assist in prolonging the circulation time as certain proteins like dysopsonins which shield the nanoparticle form rapid clearance, allowing for a better performance in vivo.

### Encapsulation efficiency and drug loading capacity

Encapsulation efficiencies of all five formulations ranged between 96.7% − 97% whilst the drug loading capacities ranged from 19.8%-23.9% as seen in Table 6. Whilst the encapsulation efficiencies were extremely high, the drug loading capacities exhibited by the lipid nanoparticles is significantly lower. Solid lipid nanoparticles generally exhibit a high encapsulation and a low drug loading capacity as seen on these studies [57–59]. The low drug loading capacity can be explained by the crystalline nature of the inner core of the solid lipid nanoparticle which tends to reduce the capability of drug incorporation [60, 61].

**Table 6:** Encapsulation efficiencies (EE%) and drug loading capacities (DL%) of the five solid lipid nanoparticle formulations ±SD (*n* = 3)

Formulation	EE%	DL%
SLN-1	96.70 ± 0.0190	21.3 ± 0.04
SLN-2	97.00 ± 0.0115	19.8 ± 0.12
SLN-3	96.96 ± 0.0055	27.2 ± 0.18
SLN-4	96.96 ± 0.0125	23.1 ± 0.06
SLN-5	96.81 ± 0.0150	23.9 ± 0.09

### Drug leakage from CTX-SLN and FBS-CTX-SLN

**Fig. 9:**
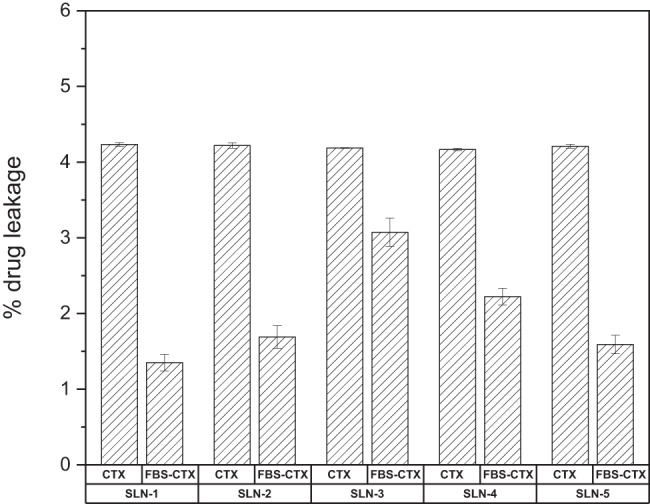
Percentage drug leakage in both CTX-SLN and FBS-CTX-SLN

Whilst solid lipid nanoparticles/nanocarriers are popular in drug delivery, a drawback that is drug leakage from the nanoparticle/nanocarrier still exists [57, 62]. As seen in Fig. 9, an average drug leakage of 4% is observed in CTX-SLN formulations. After the formation of the protein corona, drug leakage was observed to reduce by almost half. The aggregation of protein around the surface of the nanolipid blocks the release of the drug significantly. SLN-3 exhibited the smallest protein corona hence the drug leakage difference reduced by a mere 33% supporting the theory that protein corona does have an effect on drug release.

### Cumulative drug release and drug release kinetics

**Fig. 10:**
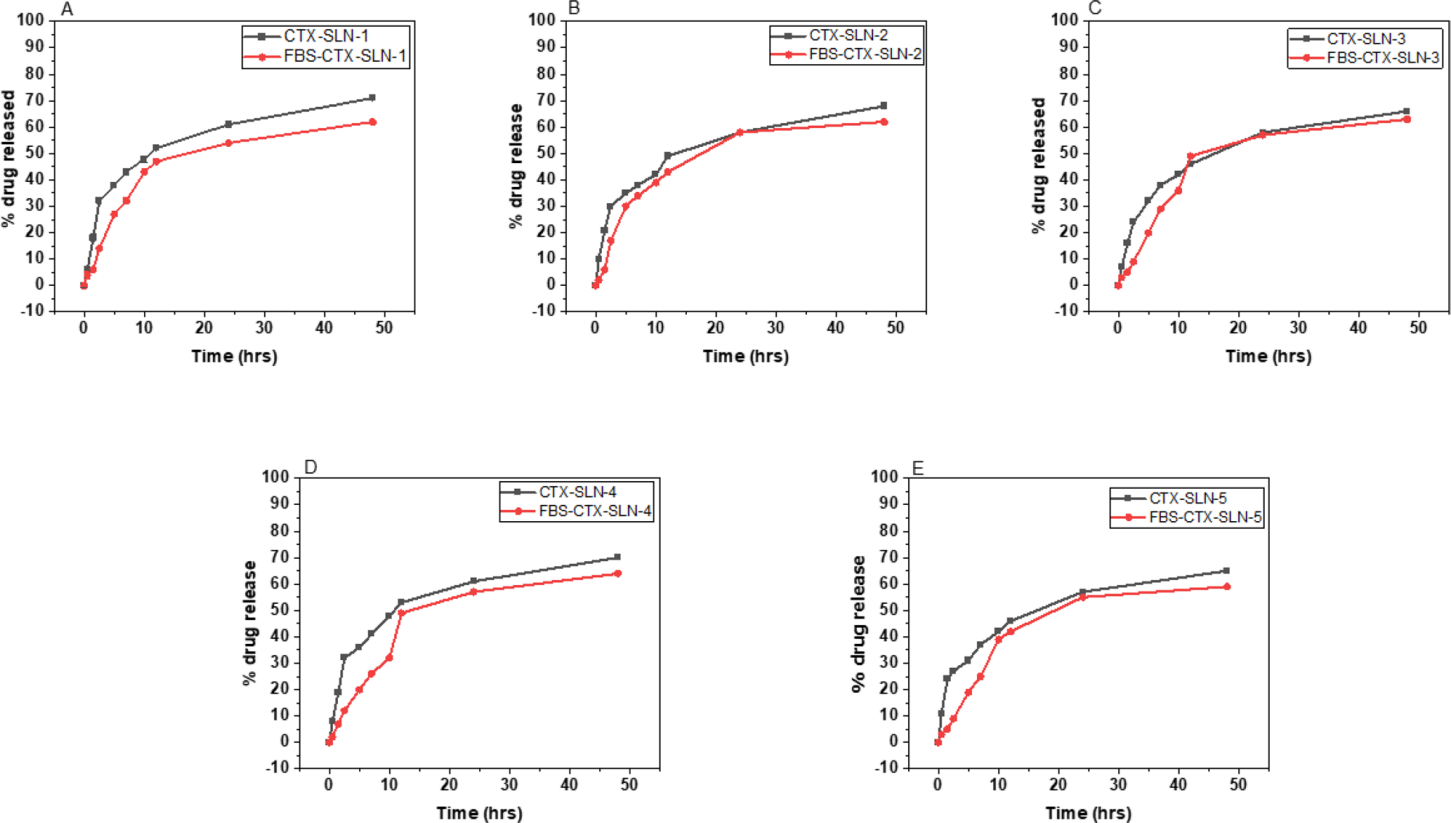
A contrast between the cumulative drug release over 48 hours of all five formulations of ceftriaxone loaded solid lipid nanoparticles, with protein corona (FBS-CTX-SLN) and without protein corona (CTX-SLN). Drug release is shown as follows: (**A**)-SLN-1; (**B**)-SLN-2; (**C**)-SLN-3; (**D**)-SLN-4; (**E**)-SLN-5

The cumulative drug release of ceftriaxone from the solid lipid nanoparticles was studied for a period of 48 hours. In CTX-SLN, the drug release was observed to have more of a burst release as compared to FBs-CTX-SLN. The drug release percentages were 71 ± 0.97%, 68 ± 0.87%, 66 ± 0.94%, 70 ± 0.76% and 65 ± 1.02% for CTX-SLN-1, CTX-SLN-2, CTX-SLN-3, CTX-SLN-4 and CTX-SLN-5 as depicted in Fig. 10. On the other hand, the drug release percentages were 62 ± 0.89%, 62 ± 0.95%, 63 ± 0.98%, 64 ± 1.05% and 59 ± 0.79% for FBS-CTX-SLN-1, FBS-CTX-SLN-2, FBS-CTX-SLN-3, FBS-CTX-SLN-4 and FBS-CTX-SLN-5 respectively. Across all formulations, the presence of the protein corona was associated with a significant reduction in cumulative drug release (*p* < 0.05). However, the key similarity in the general release profile can be noted, indicating that the amount rather than the mechanism of drug release is affected by the presence of the protein corona. Drug release in solid lipid nanoparticles occurs via diffusion from the matrix and ultimately by erosion in vivo [63]. As the present study conducted the experiments in vitro, it can be alluded that the mode of release is primarily by diffusion, Fickian diffusion to be specific as substantiated by the release kinetics done in this study. The overall lower drug release regardless of solid lipid nanoparticle formulation is attributed the biphasic release profile occurs with solid lipid nanoparticles which is a burst profile followed by a controlled release, as observed in Fig. 10 [64]. Within the first 10 hours, approximately 50% of the drug is released followed by a gradual release (~20%) over the last 38 hours. A burst release allows for the initial purge of bacteria and the controlled release inhibits bacterial resistance and the culmination of this enhances the total drug efficacy [65, 66].

Drug release profiles from carriers like nanoparticles can be explained by various kinetic models. The different models characterize the release as concentration dependent, swelling/non-swelling with regards to polymeric materials or erosion of the matrix or carrier [67]. The equations from the models were used to plot the respective graphs and thus the correlation coefficient (R^2^) values were deduced. R^2^ values are used to determine the extent with which the regression line embodies the data. From the data in Table 7, it can be noted that the best fit models of the five were the Korsmeyer and Peppas, Hixson-Crowell and Higuchi models ( > 0.9), followed closely by the first order (0.79–0.98) and lastly the zero order (0.65–0.76) model. These fittings are exemplified by other in vitro release studies by [16, 68] where the best fitting models were also the first order, Higuchi and Korsmeyer and Peppas. Lipids in nature are distinctly insoluble in an aqueous medium meaning the drug release occurs via diffusion from the lipid matrix. Zero order models are primarily the fit for studies that use lowly water soluble drugs of which ceftriaxone is readily soluble on water [69]. The low R^2^ values achieved in the zero-order model confirm this. The release kinetics of ceftriaxone loaded solid lipid nanoparticles with (FBS-CTX-SLN) and without (CTX-SLN) the protein corona were both studied. The *n* values of the Korsmeyer and Peppas being less than 0.45 represents a Fickian mode of drug release as confirmed by [70]. The fitting to the Hixson-Crowell model suggest that as time increases, there is a change in surface area of the delivery agent and this model holds true for monodispersed spheroidal materials [3]. Lastly, the Higuchi model is applicable to the release of water soluble drugs [71]. Per formulation, per model, there was an unambiguous difference in the release kinetics between a nanolipid with the protein corona and that without. This is indicative of the fact that the presence of the protein corona does not affect the manner of drug release.

**Table 7:** Kinetics of drug release of CTX-SLN and FBS-CTX-SLN (all five formulations)

Formulation	R^2^				
	Zero Order	First Order	Korsmeyer and Peppas	Hixson-Crowell	Higuchi
CTX-SLN-1	0.6513	0.8145	0.9577	0.9847	0.8951
FBS-CTX-SLN-1	0.6920	0.7939	0.9534	0.9929	0.9061
CTX-SLN-2	0.6981	0.8445	0.9356	0.9858	0.9257
FBS-CTX-SLN-2	0.6996	0.8033	0.9692	0.9926	0.9144
CTX-SLN-3	0.7091	0.8382	0.9620	0.9884	0.9346
FBS-CTX-SLN-3	0.7341	0.8179	0.9247	0.9931	0.9127
CTX-SLN-4	0.6563	0.8091	0.9490	0.9858	0.9005
FBS-CTX-SLN-4	0.7632	0.9832	0.9239	0.9926	0.946
CTX-SLN-5	0.7029	0.8359	0.9223	0.9878	0.9291
FBS-CTX-SLN-5	0.7364	0.8116	0.9269	0.9943	0.9164

### Kirby-Bauer test

**Fig. 11:**
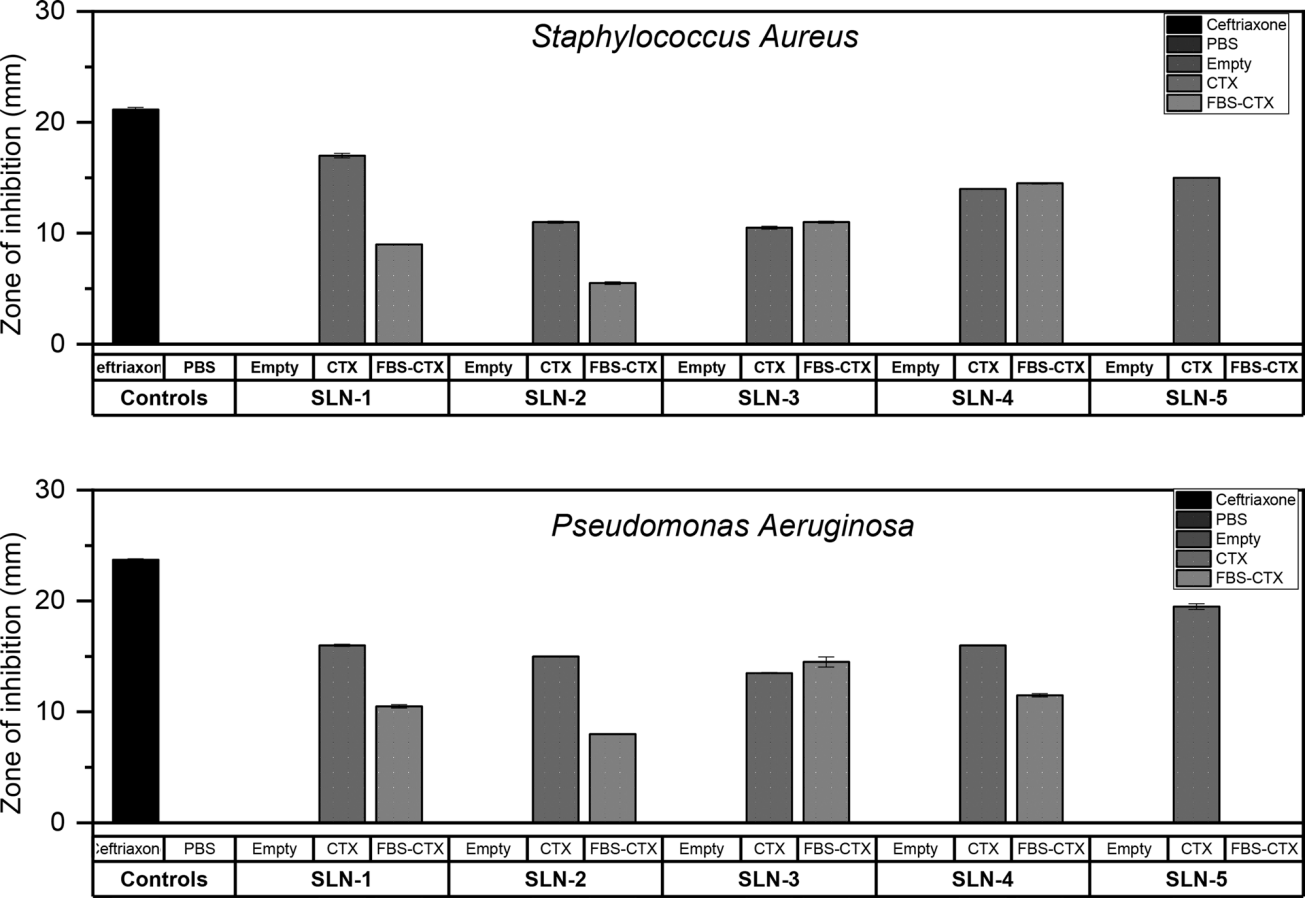
A graphical representation of the zones of inhibition by the SLN formulations. ‘Empty’ denotes SLN formulations without ceftriaxone

**Fig. 12:**
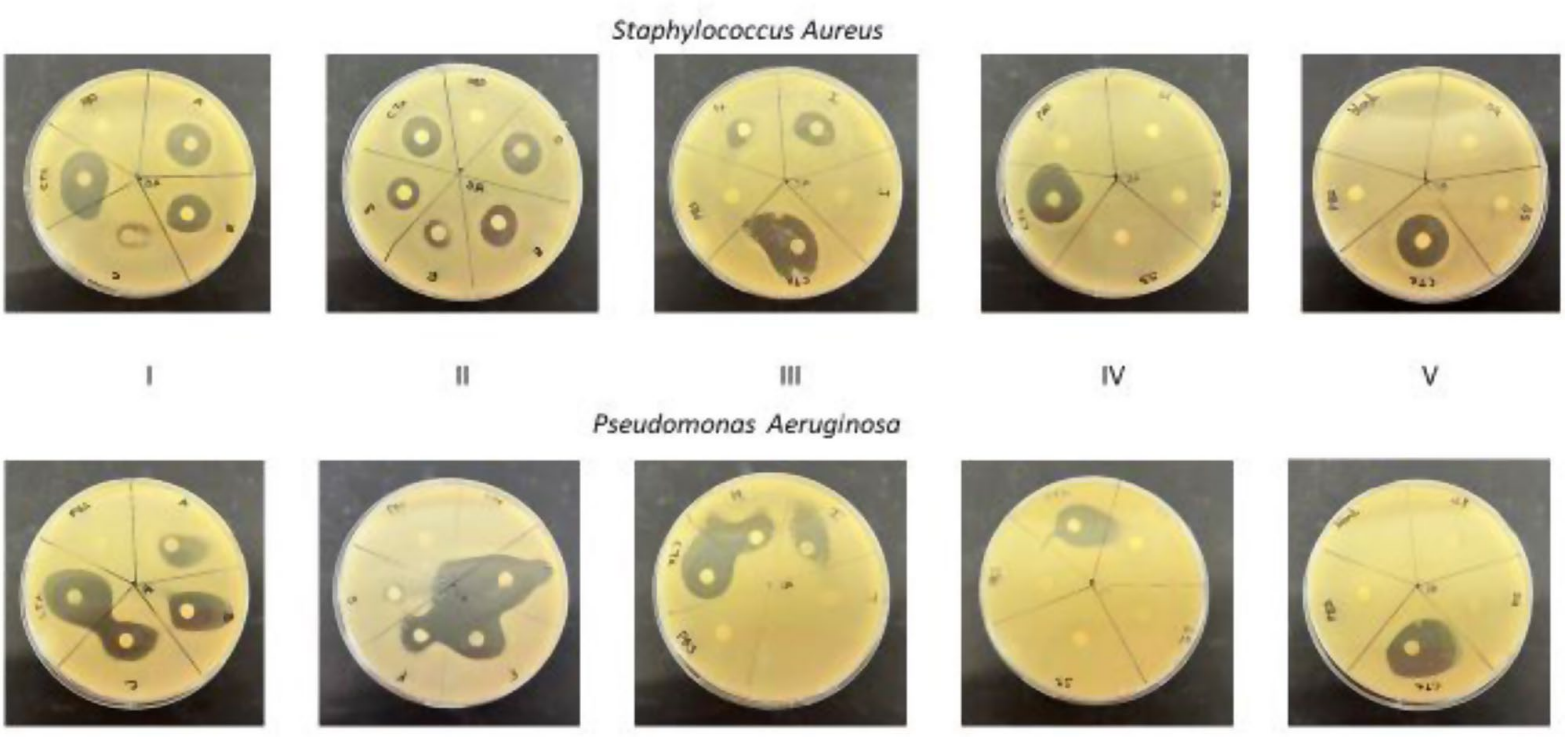
Images of the zones of inhibition for both bacterial strains. Petri dishes were organized in columns with similarly labelled fifths for each bacterial strain follows: Column I→A, B, C, CTX and PBS; Columns II → D, E, F, G, CTX and PBS; Column III → H, I, J, CTX and PBS; Column IV → S1, S2, S3, CTX and PBS; Column V → S4, S5, blank, CTX and PBS. The labels on the petri dishes are to be interpreted as follows: CTX-ceftriaxone, PBS-phosphate buffer saline solution, A→FBS-CTX-SLN-1, B→FBS-CTX-SLN-2, C→FBS-CTX-SLN-3, D→FBS-CTX-SLN-4, E→FBS-CTX-SLN-5, F→CTX-SLN-1, G→CTX-SLN-2, H→CTX-SLN-3, I→CTX-SLN-4, J-CTX-SLN-5, S1-S5 → empty (drug unloaded SLN-1-SLN-5 formulations)

The effect of the presence of the protein corona on the drug delivery potential of solid lipid nanoparticles synthesized with two surfactants was investigated on *Staphylococcus Aureus* and *Pseudomonas Aeruginosa.* The results of the test are shown in Figs. 11 and 12. Overall, the solid lipid nanoparticles without drug (SLN) showed no bactericidal effect on both strains of the bacteria. Placebo solid lipid nanoparticles have been demonstrated to show no or negligible zones of inhibition [72, 73]. Free drug in the form of 10 mg/ml of ceftriaxone was used as a positive control exhibited a clear 21.14 mm for *Staphylococcus Aureus* and 23.71 mm for *Pseudomonas Aeruginosa.* As expected, there were disparages in the cleared zones in the CTX-SLN and FBS-CTX-SLN formulations. CTX –SLN-1 has showed the largest clear zone at 17 mm whilst CTX-SLN-3 showed the smallest clear zone at 10.3 mm for *Staphylococcus Aureus.* Similar results were shown for *Pseudomonas Aeruginosa* with zones measuring 16 mm and 13.5 mm respectively. The effect of the protein corona and drug delivery is demonstrated particularly in CTX-SLN-1 and FBS-CTX-SLN-1 for both bacterial strains with an 8 mm and 5.5 mm decrease in both *Staphylococcus Aureus* and *Pseudomonas Aeruginosa* respectively. A more vivid decrease was demonstrated by FBS-CTX-SLN-2 and FBS-CTX-SLN-5 where the presence of the protein corona severely affected the amount of drug release reducing the zones by 7 mm and 19.5 mm respectively for *Pseudomonas Aeruginosa.* Overall, the presence of the biomolecular protein corona is seen to hinder the bactericidal effect of the drug loaded solid lipid nanoparticles. The negative effect of the protein corona on nanoparticle activity has been noted in several studies for example, it hindered and delayed the bactericidal effect of nanolipid carriers against *H. Pylori* and *E. Coli* [74] and negatively affected the in vivo drug delivery of doxorubicin [75]. The zones of inhibition were more prominent in *Pseudomonas Aeruginosa* than in *Staphylococcus Aureus* indicating that overall, the gram-negative bacteria appeared more sensitive to the antibiotic in this study. Interestingly, FBS-CTX-SLN-5 appeared to have no effect on both the bacteria showing a 0 mm zone of inhibition. In this investigation, the SLN-5 formulation is noted to have the highest protein corona after 24 h as seen in Fig. 8.

## Conclusion

In conclusion, the study confirmed the presence of the protein corona on stearic acid nanoparticles. The classic exchange of proteins on the nanoparticle surface between soft corona and hard corona was evidenced through both the UV-Vis spectroscopic analysis and Bradford Assay quantification. A clear relationship between nanoparticle surface charge and protein adsorption was established whereby nanoparticles with more negative zeta potential adsorbed greater quantities of protein. Prolonged incubation with foetal bovine serum (FBS) resulted in a gradual increase in the negative surface charge of the SLNs, as observed via zeta potential measurements. The role of SDS was found to be dual: while contributing to nanoparticle stabilization, higher SDS concentrations diminished overall protein corona formation, as indicated by reduced protein content measurements. Complementary in silico analysis supported these findings by elucidating potential binding interactions between proteins and the nanoparticle surface. Contingent on the purpose of the protein corona, surfactants can be used to either promote or reduce the presence of the protein corona. Tying into the findings of other studies, protein corona formation does occur on this particularly synthesized lipid nanoparticle and does affect drug release. Although the protein corona initially attenuated premature drug leakage, it ultimately altered the overall release profile. Future work should focus on proteomic characterization of the adsorbed corona components to gain mechanistic insights into their biological roles. This would provide a foundation for advancing targeted drug delivery strategies through deliberate manipulation of protein-nanoparticle interactions.

## Data Availability

Not Applicable.

## Abbreviations

SLN: Solid lipid nanoparticles
SA: Stearic acid
SDS: Sodium dodecyl sulphate
CTX: Ceftriaxone
FBS: Foetal bovine serum
BSA: Bovine serum albumin
PBS: Phosphate buffer saline
